# Statistical Optimization of Sustained Release Venlafaxine HCI Wax Matrix Tablet

**DOI:** 10.4103/0250-474X.44596

**Published:** 2008

**Authors:** M. R. Bhalekar, A. R. Madgulkar, D. D. Sheladiya, S. J. Kshirsagar, N. D. Wable, S. S. Desale

**Affiliations:** AISSMS College of Pharmacy, Kennedy Road, Near RTO, Pune-411 001, India

**Keywords:** Venlafaxine HCl, bees wax, carnauba wax, sustained release, factorial design, response surface

## Abstract

The purpose of this research was to prepare a sustained release drug delivery system of venlafaxine hydrochloride by using a wax matrix system. The effects of bees wax and carnauba wax on drug release profile was investigated. A 3^2^ full factorial design was applied to systemically optimize the drug release profile. Amounts of carnauba wax (X_1_) and bees wax (X_2_) were selected as independent variables and release after 12 h and time required for 50% (t_50_) drug release were selected as dependent variables. A mathematical model was generated for each response parameter. Both waxes retarded release after 12 h and increases the t_50_ but bees wax showed significant influence. The drug release pattern for all the formulation combinations was found to be approaching Peppas kinetic model. Suitable combination of two waxes provided fairly good regulated release profile. The response surfaces and contour plots for each response parameter are presented for further interpretation of the results. The optimum formulations were chosen and their predicted results found to be in close agreement with experimental findings.

Venlafaxine is a unique antidepressant, and is referred to as a serotonin-norepinephrine-dopamine reuptake inhibitor^1^,^2^. It works by blocking the transporter “reuptake” proteins for key neurotransmitters affecting mood, thereby leaving more active neurotransmitter in the synapse. The neurotransmitters affected are serotonin (5-hydroxytryptamine) and norepinephrine (noradrenalin). It is widely prescribed for the treatment of depression, depression with associated symptoms of anxiety, generalized anxiety disorder, and social anxiety disorder. The recommended oral dosages of venlafaxine hydrochloride are typically in the range of 75 to 225 mg per day. Because of its relatively short half-life of 5 h, venlafaxine should be administered in divided dosages throughout the day^3^.

Hydrophobic wax matrix systems are being widely used in oral controlled drug delivery because of their flexibility to obtain a desirable drug release profile, cost-effectiveness, and broad regulatory acceptance^4^. Factorial design is an optimization technique, where all the factors are studied in all possible combinations. This technique is considered most efficient in estimating the influence of individual variables (main effects) and their interaction using minimum experimentation. A Factorial Design for two factors at three levels each 3^2^ is considered identical to a two factor composite design^7^.

## MATERIALS AND METHODS

Venlafaxine HCl was obtained from Torrent Pharmaceuticals Pvt. Ltd. (Ahemadabad, India), Talc powder by Cosmo Chem. (Pune, India), and lactose, from M/s Loba Chemie Ltd. (Mumbai, India) were procured from commercial sources. All other chemicals used in the study were of analytical grade.

### Drug-excipient compatibility studies:

Drug-excipient compatibility studies were done by Fourier Transform Infrared Spectroscopy. The drug with other excipients like carnauba wax, bees wax and Eudragit L 100 (on a 1:1 ratio) were subjected to storage at room temperature and elevated temperature in stability chamber at 45°/75% RH for three month. After three month the samples were taken and IR spectrum of samples were recorded with FTIR spectrometer (460 Plus, Jasco).

### Preparation of wax matrix tablets:

The preliminary study was done by using various waxes such as compritol, precirol, carnauba wax, bees wax and stearic acid. From the preliminary study bees wax and carnauba wax were selected for further study. The bees wax was selected for its retardant effect and carnauba wax to provide mechanical strength to the matrix.

The waxes were molten and then required quantity of drug (venlafaxine HCl) was slowly added to the molten wax. After cooling, the mass was subjected to granulation by passing through the sieve no 16. Granules were mixed with lactose and talc and blend was compressed into flat-faced tablets (200 mg, 8 mm diameter) using a Rimek Mini Press-II MT tablet machine (Karnawati Eng. Ltd., Mehsana, India) to achieve a tablet thickness of 1.5±0.1 mm^8^–^10^.

Table 1 lists the composition of different formulations prepared by using varying amounts of bees wax, carnauba wax and lactose along with a fixed quantity of talc.

**TABLE 1:** COMPOSITION OF WAX MATRIX TABLET OF VENLAFAXINE HCL

Ingredients	Quantity (mg)
Venlafaxine	37.5
Bees wax	18.75–56.25
Carnauba wax	18.75–56.25
Talc	5
Lactose	q.s.

### Factorial Design:

A 3² full FD was constructed where the amounts of Carnauba wax (X_1_) and bees wax (X_2_) were selected as the factors. The levels of the two factors were selected on the basis of the preliminary studies carried out before implementing the experimental design^11^,^12^. All other formulations and processing variables were kept invariant throughout the study. Table 2 summarizes the experimental runs, their factor combinations, and the translation of the coded levels to the experimental units.

**TABLE 2:** A 3^2^ FULL FACTORIAL EXPERIMENTAL DESIGN LAYOUT

Coded factor level	

X1	X2
-1	-1
-1	0
-1	1
0	-1
0	0
0	1
1	-1
1	0
1	1

*X1 indicates amount of carnauba wax (mg); X2 amount of bees wax. Translation of coded levels in actual level is as follows, For -1, actual level of X1 and X2 is 18.75; For 0, actual level of X1 and X2 is 37.5; and For 1, actual level of X1 and X2 is 56.25.

### Physical evaluation:

Ten tablets from each batch were evaluated for uniformity in tablet weight and thickness. Tablets from each batch were examined for friability using a Roche-type friabilator (Tropical Equipment Pvt. Ltd., Mumbai, India) and hardness using a Monsanto-type hardness tester (Campbell, Mumbai, India)^13^,^14^.

### *In Vitro* Release Study^15^:

Drug release studies (n=3) were conducted for all the formulation combinations using dissolution test apparatus (Veego, DA-6D USP Standard). Distilled water (900 ml) was taken as the release medium at 100 rpm and 37±1° employing USP II paddle method (Apparatus 2). Aliquots of small samples were periodically withdrawn and the sample volume replaced with an equal volume of fresh dissolution medium. The samples were analyzed spectrophotometrically at 224 nm.

### Data Analysis:

The data obtained from dissolution kinetics studies were analyzed using PCP Disso v2.08 software developed by Poona College of Pharmacy, Pune. The computed values of kinetic constant (k) and diffusional release exponent (n) were calculated using logarithmic transformation of the relationship proposed by Korsmeyer, which was *Log( _t_/ ) Logk nLogt* (Eqn. 1), where M_t_/M_∞_ is the fraction of drug released at time t. The values of t_50%_ were calculated by MS-Excel on computers.

Various computations for the current optimization study using RSM were carried out, employing Stat Ease Design Expert Version 7^16^. Statistical second-order model including interaction and polynomial terms were generated for all the response variables. The general form of the model is, $Y={\text{β}}_{0}+{\text{β}}_{1}{\text{X}}_{1}+{\text{β}}_{2}{\text{X}}_{2}{+\text{β}}_{3}{\text{X}}_{1}{\text{X}}_{2}+{\text{β}}_{4}{\text{X}}_{1}^{2}+{\text{β}}_{5}{\text{X}}_{2}^{2}+{\text{β}}_{6}{\text{X}}_{1}^{2}{\text{X}}_{2}+{\text{β}}_{7}{\text{X}}_{1}{\text{X}}_{2}^{2}+{\text{β}}_{8}{\text{X}}_{1}^{2}{\text{X}}_{2}^{2}$ (Eqn. 2), where β_0_ the intercept, is the arithmetic average of all quantitative outcomes of nine runs, β_1_ to β_8_ are the coefficients computed from the observed experimental values of Y, and X_1_ and X_2_ are the coded levels of the independent variable(s). The terms X_1_ X_2_ and X^2^_i_ (i= 1, 2) are the interaction and polynomial terms, respectively. The statistical validity of the polynomials was established on the basis of Yates’ ANOVA. Subsequently, feasibility as well as grid search was performed to locate the composition of optimum formulations. Also, three-dimensional response surface graphs and contour plots were drawn in MS-Excel using the output files generated by the State Ease Design Expert Version-7 software.

### Validation of Optimization Model:

Six optimum formulations were selected by intensive search, performed over the entire experimental domain, to validate the chosen experimental design and polynomial equations. The criterion for selection of optimum was primarily based on the highest possible values of the response parameters, which are released in 12 h and t_50%_. The formulations corresponding to this optimum were prepared and evaluated for various response properties. The resultant experimental data of response properties were subsequently quantitatively compared with predicted values, also linear regression plots between these, forcing the line through the origin were attempted.

## RESULTS AND DISCUSSION

FTIR spectrum shows no evidence of interaction between drug and studied excipients. All the major drug peaks (functional group) at 3669 cm^-1^ [CH stretch]; 1438 cm^-1^ [N-(CH_3_)_2_ ] and 2995 cm^-1^ [OH] were seen in subsequent spectra of drug and excipients kept together. The literature documented that significant reduction in the dose frequency can be achieved via SR drug delivery system of venlafaxine HCl^17^,^18^. Design of experiment (DOE) has been widely used in pharmaceutical field to study the effect of formulation variables and their interaction on response variable^19^.

The nine formulations were designed, using various higher and lower levels of carnauba wax and bees wax (Table 1). All the preparations of each formulation passed weight variation test; the weight variation in all the nine formulations was found to be 198.5 mg to 202.8 mg, which was within pharmacopoeial limits. The hardness was found to be between 6 to 7 kg/cm^2^. Friability of all the formulations was found to be less than 0.5%. In the current study, Table 3 shows that with the increasing amount of carnauba wax and bees wax, the release after 12 h is decreased and time taken for 50% drug release increases linearly.

**TABLE 3:** DISSOLUTION PARAMETERS FOR WAX MATRIX FORMULATIONS (N=3) PREPARED AS PER 3^2^ FACTORIAL DESIGN

Trial No.	Release after 12 h ± SD	T_50_% (h)	n	k	Model
F1	88.21±0.0384%	1.17±0.0821	0.2437	49.02	Peppas
F2	78.46±0.0496%	3.14±0.0649	0.3791	30.61	Peppas
F3	68.79±0.0787%	6.35±0.0512	0.5323	17.85	Peppas
F4	82.59±0.0632%	1.41±0.0481	0.2681	42.73	Peppas
F5	71.49±0.0618%	4.35±0.0432	0.4178	24.85	Peppas
F6	59.50±0.2212%	8.34±0.0419	0.5723	14.16	Peppas
F7	77.29±0.0361%	2.56±0.0632	0.3376	32.93	Peppas
F8	68.30±0.0590%	5.46±0.0305	0.5097	18.40	Peppas
F9	48.50±0.0651%	12.42±0.0213	0.6068	10.47	Peppas

n: Diffusional release exponent; k: Kinetic constant

Table 3 lists various dissolution kinetic parameters computed for all nine batches. In the current study, in all the nine cases studied, the n varied between 0.2437 and 0.6068. Further, the magnitudes of kinetic constant (k) ranges between 10.47 and 49.02; consequently, the value of t_50%_ varies in between 1 h 17 min to12 h 42 min according to wax content.

The mathematical relationship constructed for the studied response variables are expressed as Eqns. 2 and 3. All the polynomial equations were found to be highly statistical significant (P<0.001) as determined by ANOVA. Release 12 h= 71.46-6.89X_1_ +0.13X_2_–11.88 X_1_ X_2_–0.65 X_1_^2^– 2.34 X_2_^2^–0.17 X_1_^2^ X_2_–0.91X_1_ X_2_^2^–0.25 X_1_^2^ X_2_^2^ (Eqn. 3) and T50%= 5.26+1.71 X_1_+0.16 X_2_+3.66 X_1_ X_2_+0.36 X_1_^2^+1.11X_2_^2^+0.11 X_1_^2^ X_2_+0.23 X_1_ X_2_^2^+0.094 X_1_^2^ X_2_^2^ (Eqn. 4)

**Fig. 1:**
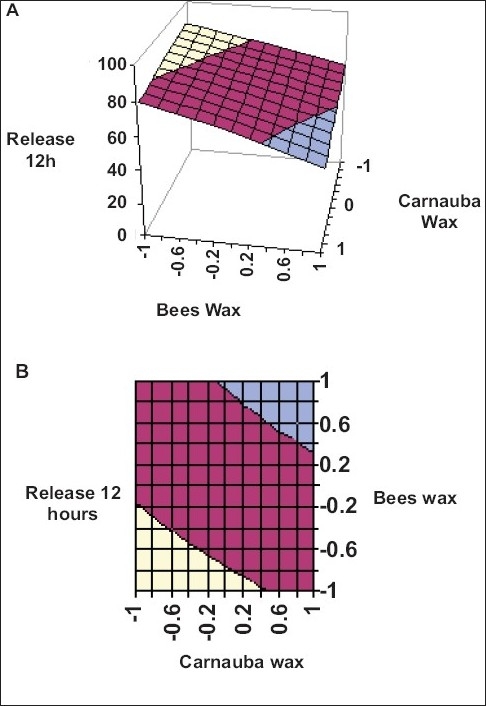
Response surface plots showing influence of carnauba wax and bees wax on percentage release in 12 h. A) Response surface plots showing influence of carnauba wax and bees wax on percentage release in 12 h for sustained release formulation of venlafaxine HCL. B) Contour plots showing relationship between various levels of carnauba wax and bees wax to attain fixed value of percentage release after 12h. 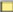 80–100% release after 12 h, 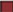 60–80% release after 12 h and 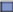 40–60% release after 12 h.

**Fig. 2:**
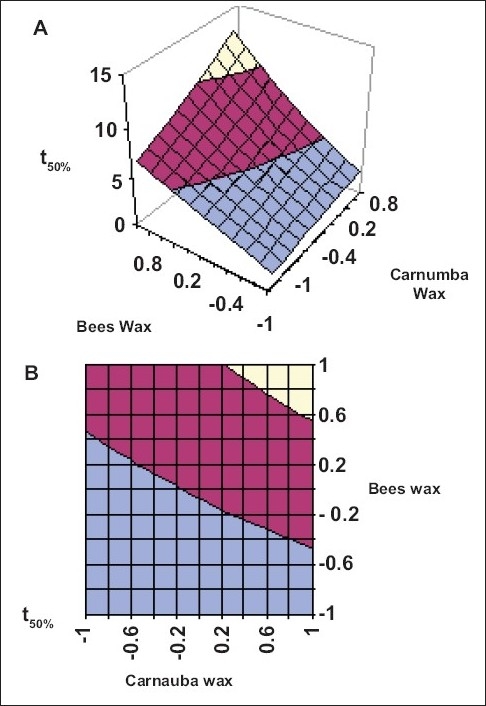
Response surface plots showing influence of carnauba wax and bees wax on time required for 50% (t_50_%) drug release (A) Response surface plots showing influence of carnauba wax and bees wax on time required for 50% (t_50_%) drug release for sustained release formulation of venlafaxine HCl. (B) Contour plot showing relationship between various levels of carnauba wax and bees wax to attain fixed value of t_50_%. 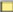 10–15 h required for 50% release of drug, 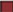 5–10 h required for 50% release of drug and 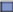 0–5 h required for 50% release of drug.

Application of two-way ANOVA based factorial analysis indicates that a high amount of carnauba wax and bees wax has a significant influence on release after 12 h and time required for 50% of drug release (P<0.001). (fig. 1), shows that release after 12 h varies in a nearly linear descending pattern with decrease in the amount of waxes. (fig. 2) also exhibits a near linear trend of t_50_%, but in ascending order. As there is no confounding of the contour lines in figs. 1 and 2, both the waxes seem to contribute independently towards drug release.

**Fig. 3:**
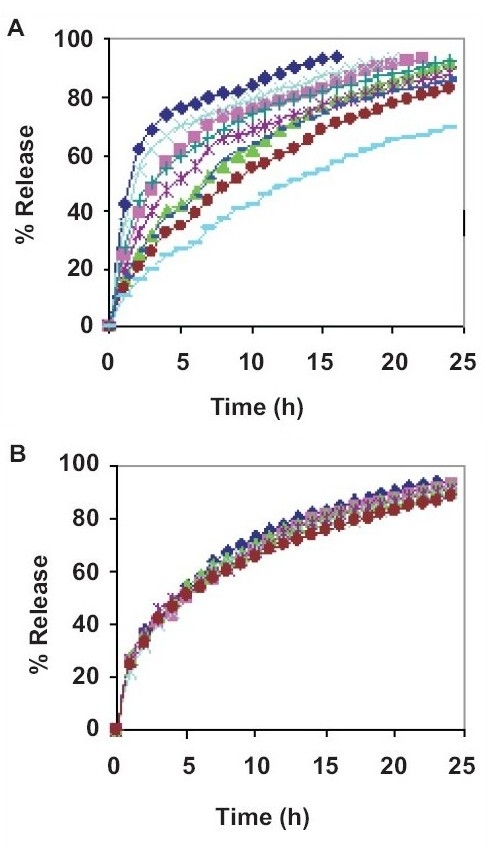
*In vitro* dissolution profile of sustained release formulations of venlafaxine HCL A) The plot shows release profile of nine formulations as per 3^2^ Factorial Design. B) The plot shows release profile of an optimum formulation. 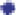 F1, 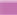 F2, 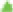 F3, 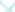 F4, 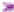 F5, 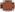 F6, 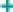 F7, 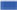 F8, 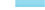 F9, 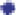 A1, 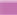 A2, 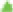 A3, 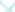 A4, 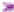 A5, 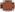 A6.

For all the six optimum formulations, the value of n ranged between 0.685 and 0.839, visibly indicating a peppas release behavior approaching. Evidently, the values of dissolution parameters had a propensity to range optimally between relatively controlled limits rather than those of the original formulations designed as per 3^2^ factorial designs. The release profile of optimum formulations shows superiority in the drug release as depicted in the figs. 3a and b.

Table 4 shows the values of observed and predicted responses using factorial design along with the percentage predicted errors for these six optimum formulations. The predicted error for the response variables ranged between -1.73 and 1.43%, with the mean±standard deviation of the percentage error being -0.2117±1.110%. Also, the linear plots between the predicted and observed responses demonstrated high of r^2^ (ranging between 0.9701 and 0.9977), indicating excellent goodness of fit. Thus, the low magnitudes of error, as well as the significant values of r^2^, designate a high prognostic ability of Response Surface Methodology (RSM).

**TABLE 4:** COMPARISON OF OBSERVED AND PREDICTED RESPONSE PARAMETERS

Formulation code	Formulation composition Carnauba/Bees wax	Response property	Experimental value	Predicted value	Percentage error
A1	18.75/41.71	Release 12 h	77.16	76.29	1.127
		T_50_%	4.40	4.31	0.681
A2	21.56/44.53	Release 12 h	73.47	73.64	-0.231
		T_50_%	5.003	4.99	0.259
A3	31.87/34.68	Release 12 h	75.88	75.20	0.896
		T_50_%	4021	4.26	-1.187
A4	34.68/37.03	Release 12 h	72.18	72.78	-0.831
		T_50_%	4076	4.83	-1.470
A5	47.81/27.65	Release 12 h	74.29	74.31	-0.026
		T_50_%	4.19	4.13	1.431
A6	52.50/29.53	Release 12 h	70.42	71.64	-1.732
		T_50_%	4.80	4.87	-1.458
Mean (±SD) of percent error					-0.2117±1.110

The results are average of three determinations. n- Diffusional release exponent, k-Kinetic constant
